# Validating and Optimizing a *Drosophila* Larval Model of Parkinson’s Synucleopathy.

**DOI:** 10.17912/micropub.biology.001592

**Published:** 2025-09-17

**Authors:** Sarah Perry, Nicholas More

**Affiliations:** 1 Biology, Austin Peay State University, Clarksville, Tennessee, United States

## Abstract

Parkinson's disease (PD) is a prevalent neurodegenerative disorder with complex genetic and environmental underpinnings. Misexpression of human alpha-synuclein (αSyn) in *Drosophila* larval dopaminergic neurons has been shown to induce PD-like phenotypes, yet this model has not been fully optimized to assess different αSyn variants and their effects on various neuronal subtypes. By expanding the use of the *Drosophila* larval model, we demonstrate that dopaminergic neurons exhibit pronounced vulnerability to αSyn, particularly to E46K and A53T variants. Additionally, we show that vitamin C effectively mitigates locomotor impairments, highlighting oxidative stress as a key contributor to αSyn-induced dysfunction.

**Figure 1. Dopaminergic neuron-specific overexpression of α-Synuclein variants results in crawling and navigational deficits:**
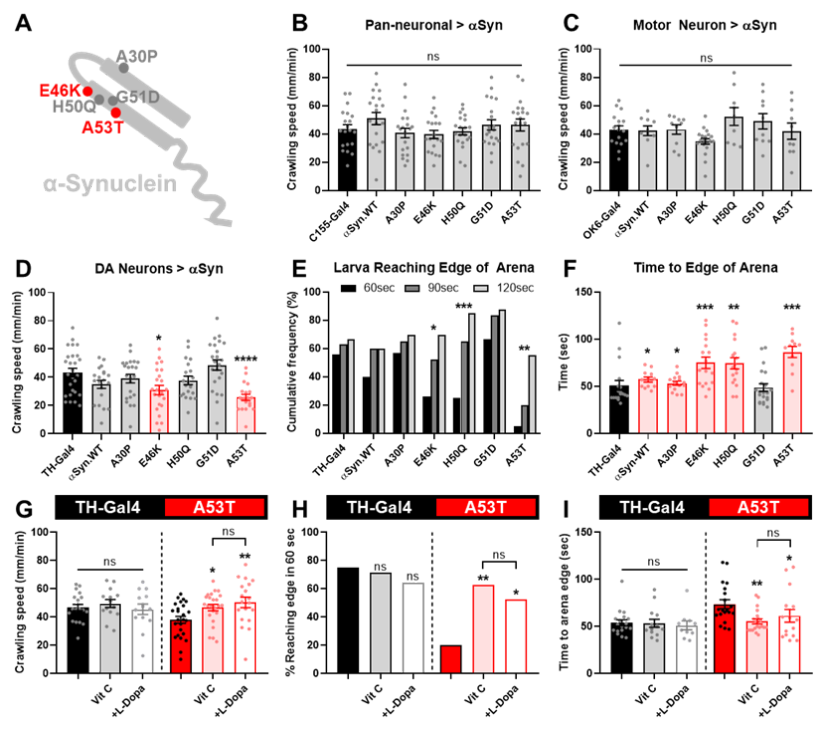
A) Schematic of human αSyn showing the positions of PD-related amino acid substitutions. B) Crawling speed of control (C155-Gal4) and pan-neuronal misexpression of αSyn (C155 > UAS-αSyn) larvae. Statistical comparisons were performed using a one-way ANOVA (N = 20). C) Crawling speed of control (OK6-Gal4) and motor neuron misexpression of αSyn (OK6 > UAS-αSyn) larvae. Statistical comparison was performed using a one-way ANOVA (N = 9-19). D) Crawling speed of control (TH-Gal4) and DAN-specific αSyn misexpression (TH > UAS-αSyn) larvae. Statistical comparisons were made between test and control genotypes using a Mann-Whitney U test (N = 20-27). E) The percentage of larvae reaching the edge of the crawling arena within 60, 90 and 120 seconds for each genotype. Statistical comparisons were performed using a Chi-squared test for independence (N = 20-27). F) The average time to edge for each genotype for all larvae who reached the edge of the crawling arena within the 2-minute trial period. Statistical comparisons between test and control groups were performed using a Mann-Whitney U test. (N = 11-21). G) Crawling speed, (H) % larvae reaching arena edge in 60 seconds and (I) average time to edge for control (TH-Gal4) and A53T (TH > UAS-αSyn.A53T) larvae reared on untreated food, food treated with Vitamin C and food treated with Vitamin C + L-Dopa. Statistical comparisons were performed using a one-way ANOVA, Mann-Whitney U test or Chi-squared test for independence (N = 14-25). (ns) p>0.05; *p<0.05; **p<0.01; ***p<0.001; ****p<0.0001

## Description

Parkinson's disease (PD) is the second most common neurodegenerative disorder, affecting approximately 0.3% of the general population. The etiology of PD involves a combination of genetic predisposition and environmental factors, making human studies inherently complex. Heritable forms of PD have been linked to mutations and duplications in the *SNCA* gene, which encodes the alpha-synuclein (αSyn) protein ( Figure 1A ) (Petrucci et al., 2016). The aggregation of αSyn is a hallmark of PD and related synucleopathies, such as Lewy body dementia (Suzuki et al., 2022). Previous research has demonstrated that misexpression of wild-type and mutant forms of αSyn in *Drosophila* is sufficient to induce PD-like phenotypes, including age-dependent dopaminergic neuron loss and motor deficits (Feany & Bender, 2000; Mohite et al., 2018; Suzuki et al., 2022). Specifically, the expression of the mutant variant A53T in larval dopaminergic neurons using the TH-Gal4 driver has been shown to induce PD-like phenotypes (Blosser et al., 2020; Varga et al., 2014). However, the larval model has not been systematically used to assess other αSyn variants or to examine synucleopathy in additional neuronal populations. To refine this model, we investigated the effects of multiple αSyn variants in different neuronal populations within the *Drosophila* larval system.

**Results**

To optimize the larval model, we expressed wild-type αSyn (αSyn.WT) and five mutant variants (A30P, E46K, H50Q, G51D, and A53T) in the larval brain using three different neuronal Gal4 drivers: C155 (pan-neuronal expression), OK6 (motor neuron-specific expression), and TH-Gal4 (dopaminergic neuron-specific expression). We then assessed locomotor function by measuring crawling speed using a standard gridline assay.

Pan-neuronal expression of αSyn variants using C155-Gal4 did not lead to significant crawling speed deficits ( Figure 1B ). This aligns with previous findings in adults, where C155-driven αSyn expression primarily results in aging-dependent phenotypes (Mohite et al., 2018). We speculated that more targeted expression of αSyn utilizing other driver lines may produce more short-term degenerative effects. Motor neuron-specific expression of αSyn using OK6-Gal4 did not cause observable crawling deficits ( Figure 1C ). The neuromuscular junction is a valuable model for studying neurodegenerative diseases at the single-synapse level, yet our findings suggest that motor neurons may be resistant to αSyn pathology or that the larval stage does not provide sufficient time for age-dependent phenotypes to manifest.

Conversely, dopaminergic neuron-specific expression of a subset of αSyn variants using TH-Gal4 produced significant locomotor impairments. Specifically, larvae expressing the E46K and A53T variants displayed reduced average crawling speed, consistent with prior findings for A53T ( Figure 1D ) (Varga et al., 2014). Healthy third-instar larvae typically travel in a straight trajectory and >50% reach the edge of a 100mm crawling arena within a 120 second trial period. In contrast, αSyn-expressing larvae showed increased reorientations and prolonged navigation times, with significantly fewer larvae reaching the edge in the E46K, H50Q, and A53T groups ( Figure 1E ). Additionally, all αSyn variants, except G51D, displayed a significant increase in the time required to reach the edge among larvae that successfully completed the trial ( Figure 1F ).

To further validate the model, we examined the effect of L-Dopa treatment, which has been previously shown to rescue A53T-induced locomotor deficits in larvae (Blosser et al., 2020). Consistent with prior studies, L-Dopa supplementation successfully restored both crawling speed and navigational ability in A53T larvae. However, to prevent oxidation and loss of potency, L-Dopa must be co-administered with an antioxidant such as vitamin C and the effect of vitamin C alone has yet to be tested (Blosser et al., 2020). Given that αSyn aggregation is known to elevate oxidative stress in dopaminergic neurons (Aryal & Lee, 2019), we hypothesized that vitamin C alone might ameliorate locomotor deficits. Indeed, vitamin C treatment was sufficient to rescue both crawling speed and navigational impairments, with no additional benefit observed from L-Dopa co-administration ( Figure 1G- I).

**Discussion**

Our findings support the utility of the *Drosophila* larval model in studying PD synucleopathy and highlight the differential effects of αSyn variants in distinct neuronal populations. Dopaminergic neurons appear particularly vulnerable to αSyn toxicity, with E46K and A53T variants exerting the strongest locomotor impairments and G51D having no effect. αSyn mutant variants are known to display a diverse range of lipid aggregation and fibril formation characteristics (Ruf et al., 2019) (Flagmeier et al., 2016). These differences may explain why some variants caused motor impairments in the larval system while others did not. For example, A53T displays elevated fibril amplification and lipid aggregation while G51D displays depressed fibril amplification and lipid aggregation (Flagmeier et al., 2016). Furthermore, vitamin C effectively mitigates locomotor deficits, suggesting that oxidative stress plays a critical role in αSyn-induced dysfunction. These results provide a refined framework for using the larval model to investigate PD pathogenesis and therapeutic interventions.

## Methods

Fly genotypes and rearing:

Flies were reared under standard conditions on cornmeal food at 25°C in a 12-hour light/dark cycle incubator. Larval density for crosses was controlled by pairing 6 females with 3-5 males and allowing the crosses to seed for 2-3 days before transferring the parents to a new tube. Wandering third instar larvae were typically observed on day 5 or 6 of culturing. Control genotypes were generated by outcrossing Gal4 females to *w1118* males, and the resulting progeny were used for experiments. Fly stocks used in this study were obstained from the Bloomington Drosophila Stock Center (BDSC): *w1118* (Perry lab stock), *C155-Gal4* (BDSC_458), *OK6-Gal4* (BDSC_64199), *TH-Gal4* (BDSC_8848), *UAS-αSyn.WT* (BDSC_8146), *UAS-A30P* (BDSC_8147), *UAS-E46K* (BDSC_80043), *UAS-H50Q* (BDSC_80045), *UAS-G51D* (BDSC_80044), and *UAS-A53T* (BDSC_8148).

Drug treatments:

Vitamin C stock solution was prepared by dissolving 1.25g ascorbic acid into 50mL deionized water. This solution was diluted 1:100 into molten food. L-Dopa was added to the Vitamin C stock solution at a concentration of 10mM and subsequently diluted 1:100 into molten food. Larvae were reared on treated food from egg to wandering third instar.

Gridline crawling assay:

Single wandering third instar larvae were placed on a 100mm 1% agarose plate placed over 5mm grid paper. The number of grid lines each larva crossed during a 2-minute trial period was manually recorded and converted into average crawling speed (mm/min). The timer was started only after the first peristaltic contraction, which was considered the first attempt at forward motion. If a larva reached the edge of the plate before the end of the 2-minute trial, the timer was stopped, and the elapsed time was recorded.
